# Effect of Sulfur Variation on the Vulcanizate Structure of Silica-Filled Styrene-Butadiene Rubber Compounds with a Sulfide–Silane Coupling Agent

**DOI:** 10.3390/polym12122815

**Published:** 2020-11-27

**Authors:** Sangwook Han, Bonyoung Gu, Sungwoo Kim, Seongrae Kim, Dalyong Mun, Koichi Morita, Donghyuk Kim, Wonho Kim

**Affiliations:** 1 Department of Polymer Science and Chemical Engineering, Pusan National University, Busandaehak-ro 63beon-gil, Geumjeong-gu, Busan 46241, Korea; sw.han@nexentire.com (S.H.); ehdgurzxc@gmail.com (D.K.); 2 Precedent Research Business Sector, Future Technology Research Institute, Nexentire, 177, Magokjungang-ro, Gangseo-gu, Seoul 07788, Korea; bon09@nexentire.com (B.G.); ksw@nexentire.com (S.K.); srkim@nexentire.com (S.K.); 10181002@nexentire.com (D.M.); morita@nexentire.com (K.M.)

**Keywords:** filler–rubber interaction, silica-filled compound, vulcanizate structure, sulfide–silane

## Abstract

The vulcanizate structure of filled compounds is affected by filler–rubber interactions (FRI) and the chemical crosslink density (CCD) of the matrix rubber. In particular, in filled compounds using a silica–silane system, FRIs due to silica–rubber coupling are a major influencing factor for the vulcanizate structure and physical properties. In this study, the effect of sulfur variation on the vulcanizate structure of silica-filled solution styrene–butadiene rubber compounds using a sulfide–silane coupling agent was studied. The vulcanizate structure according to sulfur variation was quantitatively analyzed using the swelling test and Flory–Rehner and Kraus equations. As the sulfur content increased, both FRI and the CCD increased, and it was confirmed that sulfur variation influenced the silica–rubber coupling efficiency through increased FRI. In addition, field emission scanning electron microscope images showed that increased FRI contributed to improvements in silica dispersion, abrasion resistance, and energy loss characteristics.

## 1. Introduction

The tire labeling system introduced in Europe in 2012 enforces regulations on rolling resistance (RR), wet grip (WG), and pass-by noise performance. Due to the recent tire road wear particle issue, additional regulations on wear performance are likely to be implemented. Considering only the characteristics of tread compounds, the RR performance of a passenger car radial tire (PCR) becomes more advantageous as the energy loss decreases. However, the WG performance becomes more advantageous as the energy loss increases. Therefore, most studies on PCR tire tread compounds for improving RR performance have concentrated on reducing energy loss while minimizing WG performance degradation. The introduction of the silica–silane system has significantly improved the RR performance of tread compounds, which traditionally uses carbon black as a filler, and has achieved remarkable research results complementing the trade off in WG performance caused by energy loss reduction [1]. In addition, it is known that when inorganic materials like silica form composites with organic materials, the interaction between these components increases toughness, rigidity, and the thermal stability of composites [2,3].

**Scheme 1:**
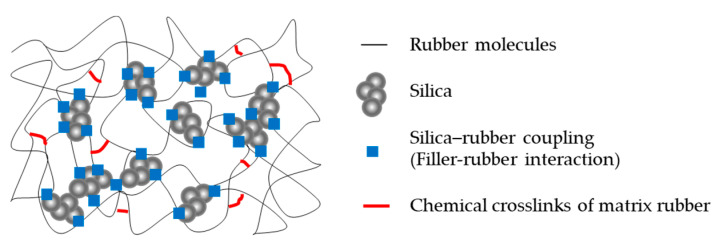
Vulcanizate structure of silica-filled compounds.

A number of recent studies have quantitatively analyzed the vulcanizate structure (total crosslink density; TCD) of silica-filled compounds using swelling tests and the Flory–Rehner and Kraus equations [4,5,6,7,8,9,10]. The TCD of silica-filled compounds has been found to reflect two factors: (1) filler–rubber interactions (FRI) due to the silica–rubber coupling, and (2) the chemical crosslink density (CCD) of the matrix rubber, as shown in Scheme 1. In addition, it was confirmed that FRI in the silica–silane system varies depending on the type of silane and the polymer (functionalized or non-functionalized); that is, FRI is changed by factors that influence the coupling reaction of silica–rubber.

In general, FRI and CCD are major influencing factors that determine the dynamic properties (e.g., RR and grip performance of compounds) and mechanical properties (e.g., tensile and wear performance). In particular, the higher the FRI, the lower the filler agglomerate in the compounds, resulting in better dispersity of the filler [5,6,7,11,12]. Reduction of filler agglomerate increases the rubber volume fraction in vulcanizate by reducing trapped rubber in agglomerates [5,6,7]; furthermore, it also impacts energy loss characteristics in a number of ways [13]. First, the decrease in the filler agglomerate decreases hysteresis in the RR region, which is the energy loss area due to the filler. Second, the increase in rubber volume fraction in the compounds increases hysteresis in the glass transition temperature (*T*_g_) region. In particular, as summer tread compounds have a *T*_g_ of approximately −10 °C, an increase in the rubber volume fraction in the compounds increases hysteresis in the WG region (0 °C). Simply, it can be observed that the higher the FRI, the better the performance balance due to better dynamic properties such as RR and WG. In addition, from the viewpoint of filler reinforcement, an increase in FRI improves mechanical properties such as modulus and wear performance [5,6,9]. Therefore, to reduce the performance tradeoff of filled compounds and secure the performance balance, it is necessary to study the compound design factors that can improve FRI.

The silica–rubber coupling reaction in the silica–silane system occurs during the mixing stage and at the vulcanization stage [14,15,16,17,18]. Luginsland [19] reported that the coupling reactivity of silane–rubber increased as the sulfur chain length of sulfide-silane increased. Hasse et al. [9] studied the coupling efficiency of an unfilled liquid polybutadiene matrix and (bis(triethoxysilylpropyl)disulfide) [TESPD] and confirmed that elemental sulfur was inserted into sulfide in the TESPD, where the coupling efficiency increased as the elemental sulfur content increased [20]. Furthermore, they proposed that the mechanism of sulfur insertion into the sulfide–silane was consistent with the well-known ZnO/CBS-accelerated vulcanization mechanism. Here, the coupling efficiency was quantitatively confirmed by analyzing the content of unreacted TESPD after a coupling reaction using high performance liquid chromatography (HPLC). However, the coupling efficiency of silica-filled compounds was indirectly predicted using the reinforcement factor (modulus ratio E_300_/E_50_). Therefore, the quantitative coupling efficiency could not be determined. Considering the above research results, sulfur variation in the silica–silane system using a sulfide-silane coupling agent is expected to be one of the factors that affects the silica–rubber coupling efficiency; that is, the FRI of silica-filled compounds is expected to change depending on the sulfur variation. However, quantitative analysis of the vulcanizate structure (FRI, CCD) of silica-filled compounds by sulfur variation has not been reported.

Taking this into account, in this study, the effect of sulfur variation on the vulcanizate structure in a silica–silane system with a sulfide–silane coupling agent was investigated. Changes in the FRI and CCD of silica-filled compounds by sulfur variation were confirmed, and their effects on the RR, WG, and mechanical properties were evaluated.

## 2. Experimental

### 2.1. Materials

Solution-SBR (SLR4630, styrene content 25%, vinyl content in butadiene 63%, 37.5 phr oil extended, Trinseo Korea Ltd., Ulsan, Korea) was used as the rubber. Silica (Ultrasil 7000GR, Evonik Korea Ltd., Seoul, Korea) with a specific surface area of 160 m^2^/g and a Brunauer–Emmett–Teller surface area (BET) of 170 m^2^/g was used as the main filler. Bis(triethoxysilylpropyl) tetrasulfide (TESPT, Si-69, Evonik Korea Ltd., Seoul, Korea) was used for the sulfide–silane. Zinc oxide (ZnO) and stearic acid were used as curing activators (both from Sigma-Aldrich Corp., Seoul, Korea). Normal sulfur (Daejung Chemicals & Metals Co. Ltd., Siheung, Korea) and the accelerator N-cyclohexyl-2-benzothiazyl sulfonamide (CBS, 98%, Tokyo Chemical Industry Co. Ltd., Tokyo, Japan), diphenyl guanidine (DPG, 98%, Tokyo Chemical Industry Co. Ltd., Tokyo, Japan), and zinc dibenzyl dithiocarbamate (ZBEC) were used as vulcanizing agents. Tetrahydrofuran (THF, 99%, Samchun Chemical Co., Seoul, Korea), n-hexane (95%, Samchun Chemical Co., Seoul, Korea), and toluene (99.5%, Samchun Chemical Co., Seoul, Korea) were used as solvents for analyzing bound rubber and vulcanizate structures. Reagents and solvents were used as received.

### 2.2. Measurements

An oscillation disk rheometer (ODR; ODR 2000, Myung Ji Co., Seoul, Korea) was used to confirm the vulcanization characteristics of the unvulcanized compound. The conditions of ODR measurements were as follows: a vibration angle of ±1° and a temperature of 160 °C. Vulcanizates were prepared using a hydraulic press at 160 °C for 1.2 times the optimum curing time (*t*_90_ × 1.2 min) to study their characteristics.

A universal testing machine (UTM; KSU-05M-C, KSU Co., Ansan, Korea) was used to measure the mechanical properties of the vulcanizates. Tensile properties such as modulus, tensile strength, and elongation at break were measured according to the ASTM D412: extension speed of 500 mm/min and a load cell of 500 N. The abrasion resistance of vulcanizates was evaluated using a William abrasion tester at a load of 1 kg and a rotation speed of 75 rpm for 10 min.

Field emission scanning electron microscopy (SEM) with energy dispersive x-ray spectroscopy (FE-SEM-EDX; JEOL Co., Tokyo, Japan) was used to confirm the degree of silica dispersion in the vulcanizates. For SEM image analysis, specimens were pretreated via etching for 2 h under an accelerating voltage of 5 kV using argon ion milling equipment (IB-19520 CCP, JEOL Co., Tokyo, Japan). The FE-SEM-EDX analysis conditions showed a surface image using a magnification of ×1000, an accelerating voltage of 5 kV, and a back scattered electron detector. The SEM image was quantitatively analyzed for silica agglomerate size and distribution using image analysis software (Inner view, Inner View Co., Seongnam, Korea).

To study the viscoelastic properties of the vulcanizates, a dynamic mechanical thermal analyzer (DMTA; EPLEXOR 500 N, GABO, Ahlden, Germany) was used. The measurement conditions of the temperature sweep were as follows: amplitude of 30 μm, frequency of 10 Hz, tension mode, 0.2% dynamic strain, and temperature sweep (−80 to 70 °C). The degree of silica agglomeration in the vulcanized state was confirmed by the change in the storage modulus under 10 Hz, 60 °C, and 0.2–5% strain sweep conditions.

To confirm the wet frictional properties, a rotational traction measuring system (RTMS; FR-7225, Ueshima, Kobe, Japan) was used to measure the direct friction coefficient between the road surface and rubber specimen. The wet friction coefficient was measured using a rubber specimen of a circular shape (diameter: 80 mm) at room temperature (25 °C) under the following conditions: load of 70 N, water flow of 5 min/L, speed of 30 km/h, and maximum slip ratio of 95%, and the asphalt road of the RTMS.

### 2.3. Preparation of Vulcanizates

The formulation of compounds used for vulcanizate structure analysis according to sulfur variation are shown in Table 1. Silica with a specific surface area of 160 m^2^/g was applied, and silica contents of 60 and 80 phr were used. To confirm the vulcanizate structure using a Kraus plot, a compound with a silica content of 40 phr was also prepared. The silane content was 8% of the silica content. The sulfur content applied in the tread compound was 0.9, 1.2, or 1.5 phr. The batch weight was adjusted to a fill factor of 55% of the intermesh mixer. Table 2 shows the mixing process used in this study. SSBR was pre-mixed for 30 s, and the silanization reaction was performed for 3 min at 145 °C after silica-silane was added. The discharge temperature of stage 1 was 145 °C after silanization. In stage 2, the final mixing state, sheeted rubber of stage 1, sulfur, and accelerators were added, mixed to 100 °C, and then discharged.

### 2.4. Analysis of Vulcanizate Structure

Vulcanized specimens with dimensions of 10 mm × 10 mm × 2 mm were prepared to study the vulcanizate structure. These specimens were treated with THF and n-hexane at room temperature (20–25 °C) for two days to extract organic materials contained in the vulcanizates. After extraction, the specimens were dried in a 40 °C vacuum oven for two days. To analyze the TCD of the vulcanizates, dried specimens without organic material were treated with toluene at room temperature (20–25 °C) for 24 h. After treatment, the TCD was measured based on the weight difference of the specimen before and after swelling. The value of the volume fraction of vulcanizate in the swollen gel (*v*_r_), for the Flory–Rehner equation, was calculated using Equation (1):

(1) $$v_{r}=\frac{\frac{w_{dry}-w_{filler}}{\rho_{rubber}}}{\frac{w_{dry}-w_{filler}}{\rho_{rubber}}+\frac{w_{swollen}-w_{dry}}{\rho_{solvent}}},$$

where $w_{dry}$ is the weight of the dry sample; $w_{filler}$ is the weight of filler in the dry sample; $w_{swollen}$ is the weight of the swollen sample; $\rho_{rubber}$ is the density of rubber; and $\rho_{solvent}$ is the density of the solvent.

## 3. Results and Discussion

### 3.1. Vulcanization Characteristics

Vulcanization behavior according to sulfur variation was confirmed through ODR evaluation. As shown in Table 3, when the silica content was the same, *t*_10_ (i.e., the cure time for 10% vulcanization) slightly decreased as the sulfur content of each compound increased; this is because increasing sulfur content is advantageous for generating active sulfurizing species in the initial stage of vulcanization [21]. However, *t*_90_, which is the cure time for 90% vulcanization, tended to be slightly longer as the TCD increased and more curing time was required. The Δ*T* (torque) increased with increasing sulfur content, which showed typical vulcanization behavior according to sulfur variation [21]. The TCD of the filled compounds is affected by the FRI and CCD [4,5,6,7,8,9,10,22]. This means that because sulfur variation in a silica–silane system with a sulfide–silane coupling agent is one of the factors affecting the silica–rubber coupling efficiency, FRI is also expected to change. Therefore, vulcanizate structure analysis was necessary to determine whether the cause of the increase in TCD was the increase in CCD or the increase in FRI.

### 3.2. Analysis of Vulcanizate Structure

**Scheme 2:**
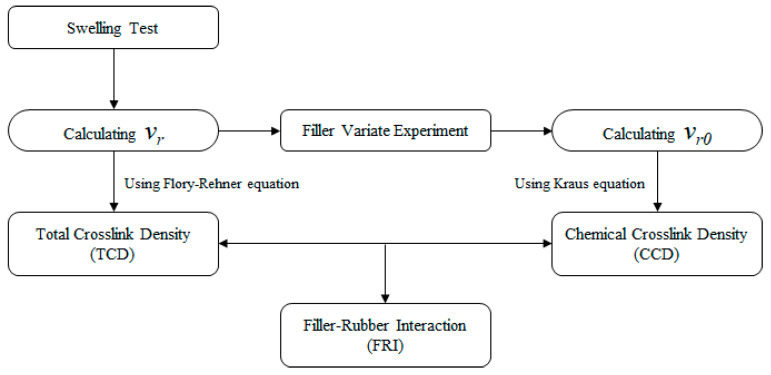
Process for analyzing vulcanizate structures. Reproduced with permission from [4]. Copyright 2017 Taylor & Francis.

**Scheme 3:**
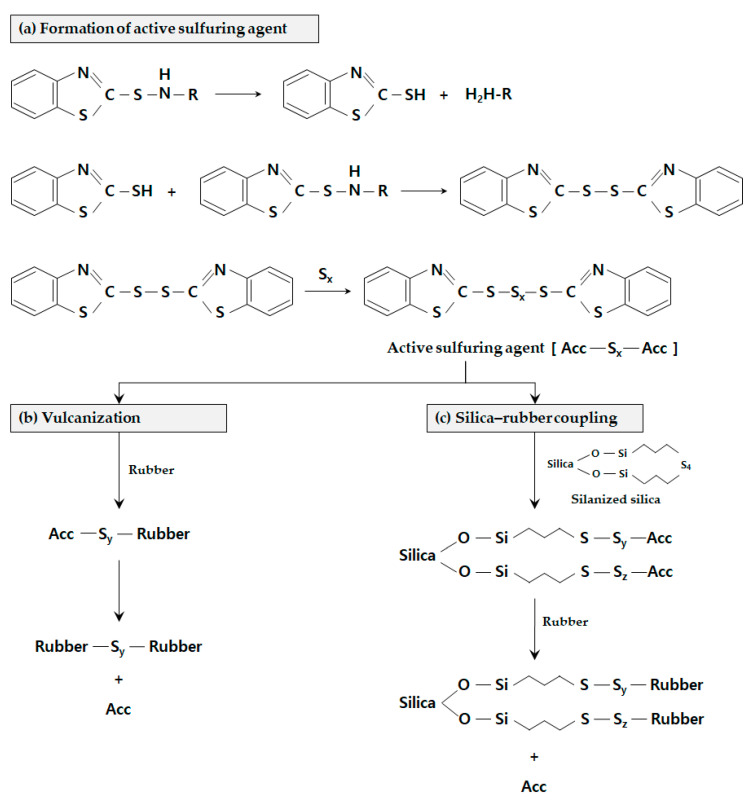
Suggested mechanisms of vulcanization and silica–rubber coupling.

The vulcanizate structure of the silica-filled compounds according to sulfur variation was confirmed using the method proposed by Lee [4] and Ahn [5,6]. The TCD was calculated using the Flory–Rehner equation [23] through a swelling experiment. The FRI, due to the silica–rubber coupling reaction, was calculated using the difference between the TCD and CCD calculated by the Kraus equation [9,24]. Here, the change in FRI at the same silica content could be interpreted as the coupling efficiency according to sulfur variation. The experimental procedure used to analyze the vulcanizate structure is shown in Scheme 2. For CCD calculation, an additional silica compound (40 phr) was prepared, and the change in the vulcanizate volume fraction in the swollen state according to the silica volume fraction (φ_f_) was confirmed. Table 4 shows the changes in TCD, CCD, and FRI of vulcanizates according to sulfur variation. When the sulfur content was the same, the TCD increased as the silica content increased, which was attributed to the increase in FRI. Here, it is considered that the FRI increased because the coupling between the silica and rubber increased with increased silica content. When the silica content remained the same, the TCD increased as the sulfur content increased, which was attributed to the increase in both FRI and CCD. Here, the increase in FRI is an interesting result because with an increasing sulfur content, the coupling efficiency in the silica–rubber increases. This is a quantitative confirmation that in silica-filled compounds, the FRI increases as the sulfur content increases, similar to the results of Hasse [11], who studied the coupling efficiency between TESPD and rubber by sulfur variation using an unfilled model compound. Therefore, the cause of increased FRI and CCD with increasing sulfur content can be expected by the suggested mechanism shown in Scheme 3. First, the increased sulfur content increases the formation of active sulfurizing agents by mechanism (a) during the vulcanization stage. Second, these increased active sulfuring agents accelerate the crosslinking reaction of the matrix rubber and the silica–rubber coupling reaction by mechanisms (b) and (c). In other words, the increase in sulfur content is expected to increase FRI and CCD by providing sufficient active sulfuring agents to generate a crosslinking reaction of the matrix rubber and silica–rubber coupling reaction.

### 3.3. Effect of Sulfur Variation on Mechanical Properties

**Figure 1:**
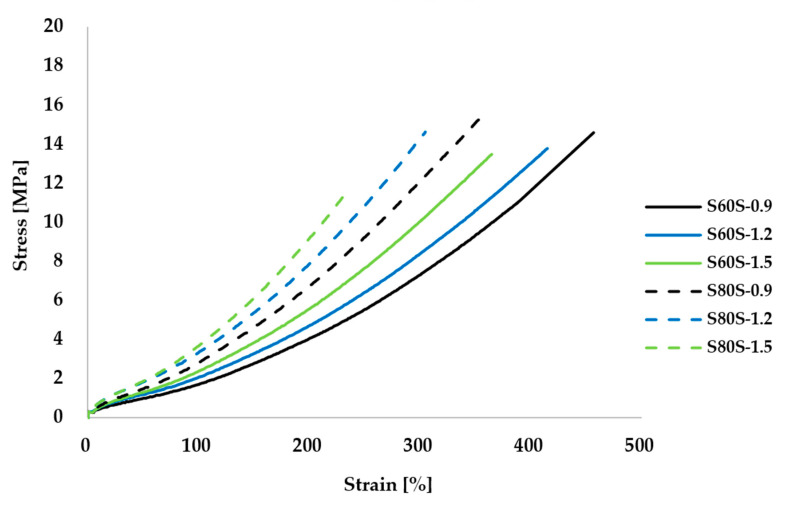
Stress–strain curves of the vulcanizates.

Table 5 and Figure 1 show the mechanical properties of silica-filled compounds according to sulfur variation. When the silica content was the same, the hardness and modulus values increased as the sulfur content increased, and the tensile strength decreased as the elongation at break decreased [11]. Hardness means stiffness at low strain, and it depends on filler content, dispersion state, and crosslink density [9,25]. Therefore, we confirmed that the increase in hardness was a result of the increase in crosslink density. Given that the FRI increased as the sulfur content increased, the possibility of increasing stiffness due to silica agglomeration was low. The modulus depends on the filler reinforcement and crosslink density; therefore, the increase in the modulus is caused by the increase in FRI and CCD due to the increased sulfur content. However, the tensile strength decreased because the elongation at break decreased when the CCD increased and the average molecule weight between the crosslink points (*M*_C_) decreased; thus, the modulus by deformation increased, however, the chain extension decreased [9,26].

Abrasion resistance, which is a measure of filler reinforcement [1,9], was improved as the silica and sulfur contents increased. The abrasion resistance improved as the silica content increased, increasing the silica volume fraction, which delayed crack propagation [9]. In addition, the improvement in abrasion resistance due to increasing sulfur content is believed to be caused by increases in FRI and CCD corresponding to the filler reinforcement, as confirmed by the vulcanizate structure result.

### 3.4. Filler Dispersity of Vulcanizates using FE-SEM-EDX Analysis

**Figure 2:**
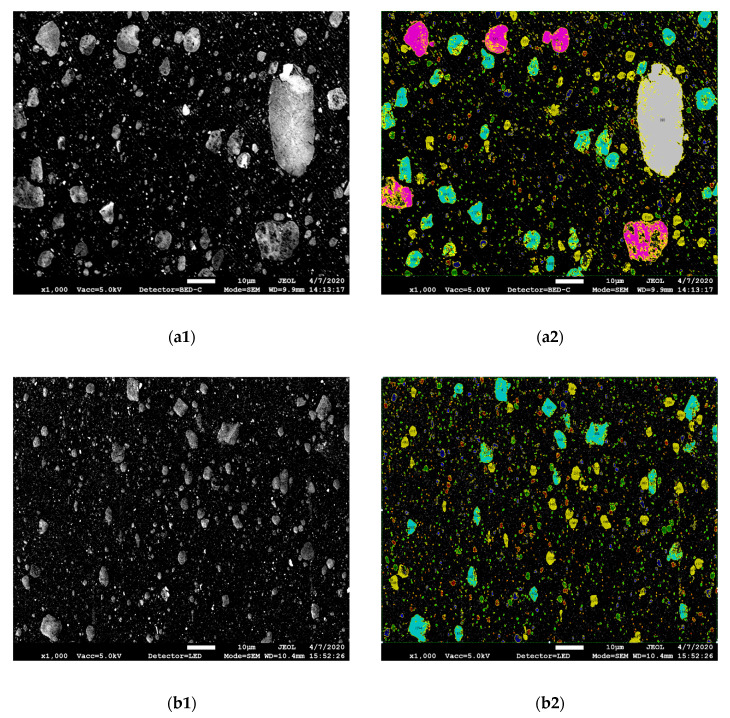
Field emission scanning electron microscope (FE-SEM) images of vulcanizates: (**a1**) S60S-0.9 (×1000), (**a2**) treated image of **a1**, (**b1**) S60S-1.5 (×1000), and (**b2**) treated image of **b1**.

Field emission scanning electron microscopy with energy dispersive x-ray spectroscopy (FE-SEM-EDX) analysis was performed to confirm the degree of silica dispersion in the vulcanized state according to sulfur variation. The specimens used for SEM image analysis were pretreated using an ion beam etching system. The measured SEM images were analyzed for silica agglomerate size and distribution using imaging analysis software. Figure 2 shows the silica dispersion by sulfur content. Each color is classified according to the size of the silica agglomerate; the range of agglomerate size and distribution are shown in Table 6. In Class 1 (0.5–1.0 µm), the S60S-1.5 compound showed a higher distribution ratio than S60S-0.9, confirming that the silica dispersion improved with increasing sulfur content. In particular, in the S60S-1.5 compound, silica agglomerates were not observed to have large sizes such as in Class 9 (20–50 µm) and Class 10 (50–100 µm), confirming that the dispersion was relatively uniform. This is in good agreement with the vulcanizate analysis; that is, the observed increases in FRI are consistent with the excellent silica dispersion in the vulcanized state at high sulfur contents.

### 3.5. Viscoelastic Properties

Energy loss characteristics under cyclic deformation conditions provide important information for predicting the dynamic characteristics of a tire. Among them, the tan δ value and loss modulus E” value, which is a loss factor in the low-temperature range (0–20 ℃), are used to predict the grip performance of the tire; the higher the value, the better the grip performance [1,27]. As the value of tan δ in the *T*_g_ region depends on the volume fraction of the rubber, the lower the filler volume fraction, the higher the value of tan δ. In addition, when the filler volume fraction was the same, the better the filler dispersion, the higher rubber volume fraction; thus, tan δ at a given *T*_g_ value increased. The *T*_g_ of the summer tread compound was approximately −10 °C; thus, the value of tan δ at 0 °C used for predicting WG performance tended to be higher with the increasing volume fraction of rubber. The value of E”, which is characteristic of viscosity, increased as the filler volume fraction increased. The value of tan δ in the high-temperature range (50–80 °C) was used to predict the RR of the tire, and it is known that a lower value of tan δ results in better fuel economy performance [9]. Wang [13] reported that the energy loss characteristics in this region resulted from the destruction and re-formation of the filler network due to periodic deformation. Tomita [28] reported that the energy loss characteristic was a result of the degree of localized deformation occurring between the fillers. Both studies showed that the energy loss characteristics in the high-temperature region depend on the state of the filler in the rubber.

**Figure 3:**
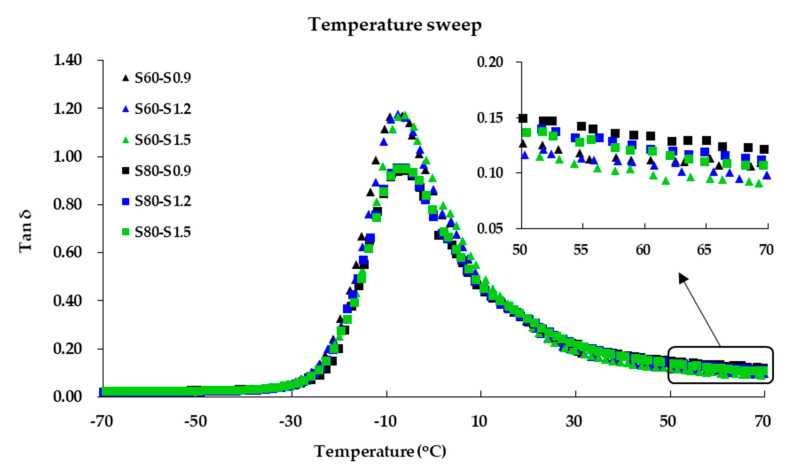
Tan δ curves measured at the temperature sweep condition.

**Figure 4:**
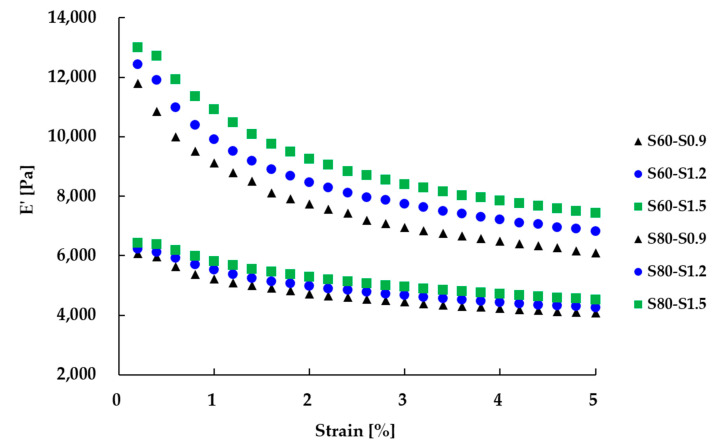
Storage modulus (*E*’) under different strain conditions at 60 ℃.

The temperature sweep results (Table 7, Figure 3) showed the energy loss characteristics of the silica-filled compounds. When the sulfur content was the same, the value of tan δ at 60 °C increased with increasing silica content. This was attributed to an increase in localized deformation between the fillers under the periodic deformation condition due to the increase in the filler volume fraction [7]. When the silica volume fraction increased, the viscosity property, *E*”, at 0 °C increased, and the energy loss property, tan δ, at 0 °C decreased, because of the decrease in the rubber volume fraction. When the silica content was the same, the value of tan δ at 60 °C tended to decrease as the sulfur content increased. This was attributed to an increase in FRI and improvement in silica dispersion, which were confirmed from the vulcanizate structure analysis and FE-SEM image analysis results. The value of E” at 0 °C tended to increase due to the increase in CCD with increasing sulfur content. The value of tan δ at 0 °C increased as the sulfur content increased, which was due to the increase in the volume fraction of the matrix rubber due to the improvement in silica dispersion resulting from the increase in FRI. The strain sweep results (Table 7, Figure 4) show the strain-dependent behavior of silica-filled compounds. As the sulfur content increased, the values of E’ at both 0.2 and 5% strains increased because of the increase in TCD. However, the value of Δ*E*’ (at 0.2–5%) showed a tendency to decrease with increasing sulfur content. Thus, as the strain increased, the degree of re-formation of the silica network was low, so silica dispersion improved. These results agreed well with the improved silica dispersion.

### 3.6. Wet Friction Coefficient of Vulcanizates Using a Rotational Traction Measuring System (RTMS)

The frictional characteristics between rubber-road surfaces are related to two stress mechanisms [29,30,31,32,33]. First, when the tread slides over roughness on the road surface, specific parts of the tread are subjected to repeated deformation, and hysteresis (energy loss) is generated by this deformation. Second, adhesion force is generated by molecular interaction (van der Waals force) between the rubber and the road surface. The grip performance of a tire is affected by the hysteresis and adhesion of the tread compound in contact with the road; the higher the hysteresis by these two mechanisms, the better the grip performance [29,30,31,32,33]. Thus, the hysteresis term can be predicted by the values of *E*” (viscosity) and tan δ (energy loss) measured using DMTA; however, more studies are required to predict the adhesion term. In particular, it is difficult to confirm because the contribution of the adhesion term to the WG is smaller than that of the dry grip. Therefore, in this study, the WG performance of the compounds was predicted using the wet friction coefficient (μ) value obtained by direct friction between the rubber and wet road surfaces using an RTMS tester.

**Figure 5:**
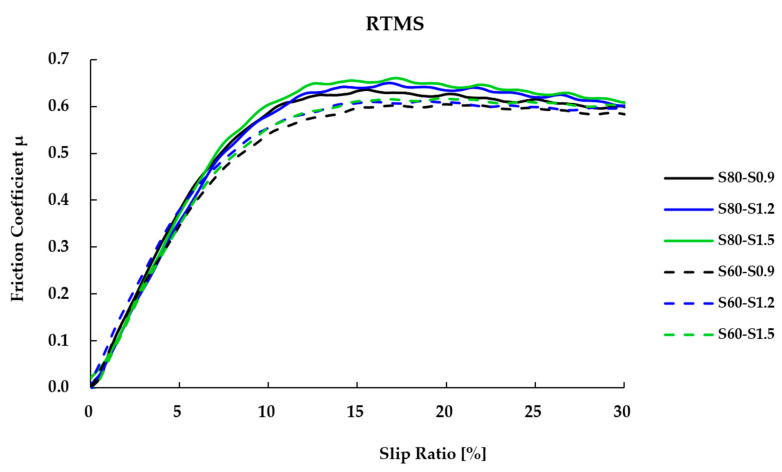
Friction coefficient μ of vulcanizates according to slip ratio.

Table 8 and Figure 5 show the measurement results of the wet μ values according to sulfur variation. When the sulfur content was the same, the value of wet μ increased as the silica content increased. This was because of an increase in the value of the hysteresis term E” at 0 °C, as confirmed by the viscoelastic properties. In addition, although not quantitatively distinguished, the increase in the interaction (adhesion term) with the wet road surface due to the increase in hydrophilic silica was the reason for the increase in wet μ. The fact that the wet μ value increased as the sulfur content increased when the silica content was the same was particularly interesting. This is because the possibility that the adhesion term of the compounds changes according to sulfur variation is low. Therefore, it is considered that the change in the wet μ value of vulcanizates according to sulfur variation is due to a change in the hysteresis term. That is, it is because both the hysteresis terms E” and tan δ at 0 ℃ increased as the sulfur content increased, as confirmed by the viscoelastic properties. Therefore, the value of wet μ improved with increasing sulfur content because of the increase in the hysteresis term (E” at 0 °C and tan δ at 0 °C) in the WG region due to the increase in FRI and CCD.

## 4. Conclusions

In this study, the effect of sulfur variation on the vulcanizate structure of silica-filled compounds using sulfide–silane was confirmed. As the sulfur content increased, the cure time (*t*_10_) at the initial stage of vulcanization slightly decreased, but *t*_90_ tended to be slightly increased due to the increase in total crosslink. The Δ*T* (torque) showed typical vulcanization behavior that increased with increasing sulfur content.

Analysis of the vulcanizate structure of silica-filled compounds according to sulfur variation was quantitatively confirmed using the Flory–Rehner and Kraus equations. TCD increased as the sulfur content increased, which was attributed to the increase in both FRI and CCD. Here, the change in FRI showed that the sulfur variation acts as a factor affecting the silica–rubber coupling reaction. Therefore, it was confirmed that sulfur variation in silica-filled compounds using a sulfide–silane coupling agent is a major influencing factor for changing FRI such as silane type and polymer type. In terms of mechanical properties, as the sulfur content increased, the hardness and modulus increased, the tensile strength decreased, elongation at break decreased, and abrasion resistance increased significantly. In particular, the large improvement in abrasion resistance was mainly due to the increases in FRI and CCD. Silica dispersion in the vulcanized state according to sulfur variation was visualized through FE-SEM-EDX image analysis. Size distribution analysis of silica agglomerates revealed the improved silica dispersion because the portion of less than 1 µm agglomerate was higher and that of larger than 20 µm agglomerates was not observed as the sulfur content increased. In terms of dynamic properties, both RR and WG performance improved because of the increased FRI and CCD as the sulfur content increased, resulting in excellent performance balance. In particular, for WG performance, the wet μ value using RTMS increased as the sulfur content increased.

Therefore, it was confirmed that sulfur variation had an effect of changing silica dispersion as well as the mechanical, and dynamic properties by changing the FRI and CCD in silica–silane systems with a sulfide–silane coupling agent.

## Figures and Tables

**Table 1:** Formulation of compounds (unit: phr).

Step	Materials	S60S-0.9	S60S-1.2	S60S-1.5	S80S-0.9	S80S-1.2	S80S-1.5
Stage 1	S-SBR	137.5	137.5	137.5	137.5	137.5	137.5
	Silica	60	60	60	80	80	80
	TESPT	4.8	4.8	4.8	6.4	6.4	6.4
	Zinc oxide	2	2	2	2	2	2
	Stearic acid	3	3	3	3	3	3
Stage 2	Sulfur	0.9	1.2	1.5	0.9	1.2	1.5
	CBS	1	1	1	1	1	1
	DPG	2	2	2	2	2	2
	ZBEC	0.2	0.2	0.2	0.2	0.2	0.2

S60, S80: silica contents; 60, 80 phr. S-0.9, S-1.2, and S-1.5: sulfur contents; 0.9, 1.2, and 1.5 phr.

**Table 2:** Mixing procedure.

Step	Time (min:seconds)	Revolutions per minute (RPM)	Action
Stage 1	0:00–0:30	15	Rubber
	0:30–1:30	30	Silica and chemicals
	1:30–1:40	30	Sweep
	1:40–5:30	Variable	Mixing and silanization (during 3 min at 145 ℃)
	5:30	Variable	Dump at 145℃ after silanization reaction
	Sheeting	-	Two roll mill
Stage 2	0:00–0:20	15	Compounds of stage 1
	0:20–1:00	30	Add sulfur, cure accelerator
	1:00–2:00	30	Extra mix and dump (under 100 ℃)
	Sheeting	-	Two roll mill

**Table 3:** Cure characteristics of vulcanizates by sulfur variation.

Compound		S60S-0.9	S60S-1.2	S60S-1.5	S80S-0.9	S80S-1.2	S80S-1.5
Cure time 10% vulcanization (t_10_)	min	3.7	3.6	3.4	3.5	3.3	3.2
Cure time 90% vulcanization (*t*_90_)	min	14.7	14.9	15.1	17.2	17.9	18.2
Torque [△*T*], *T*_max_ − *T*_min_	N·m	2.079	2.192	2.429	2.248	2.429	2.633

**Table 4:** Crosslink density of various vulcanizates.

Crosslink Density (10^−4^ mol/g)	S60S-0.9	S60S-1.2	S60S-1.5	S80S-0.9	S80S-1.2	S80S-1.5
TCD ^a^	1.14	1.26	1.39	1.44	1.64	1.75
CCD ^b^	0.55	0.63	0.70	0.55	0.63	0.70
FRI ^c^ (= a − b)	0.59	0.63	0.69	0.89	1.02	1.05

^a^ Total crosslink density; ^b^ chemical crosslink density by vulcanization reactions between vulcanization agents and matrix rubber; ^c^ crosslink density by filler-rubber interactions (FRI) in a silica–silane system (silica–rubber coupling).

**Table 5:** Mechanical properties and abrasion loss of vulcanizates by sulfur variation.

Compound	S60S-0.9	S60S-1.2	S60S-1.5	S80S-0.9	S80S-1.2	S80S-1.5
Hardness (Shore A)	57	60	61	67	68	70
*M*_100_ (MPa)	1.7	2.1	2.4	2.8	3.4	3.7
*M*_300_ (MPa)	7.3	8.4	10.1	12.1	14.3	-
Tensile strength (MPa)	14.6	13.8	13.5	15.3	14.6	11.3
Elongation at break (%)	460	420	370	350	310	230
Abrasion (weight loss, %)	10.7	7.4	6.1	0.9	0.6	0.5

**Table 6:** Agglomerate size distribution of silica in vulcanizates by sulfur variation.

Range (µm)		S60S-0.9	S60S-1.5
Class 1	0.5–1.0	18.73	34.53
Class 2	1.0–1.5	56.64	46.39
Class 3	1.5–2.0	13.21	9.28
Class 4	2.0–2.5	4.12	4.06
Class 5	2.5–3.0	1.99	1.95
Class 6	3.0–5.0	1.13	0.74
Class 7	5.0–10.0	2.06	2.00
Class 8	10.0–20.0	1.73	1.05
Class 9	20.0–50.0	0.33	-
Class 10	50.0–100.0	0.07	-

**Table 7:** Viscoelastic properties of vulcanizates by sulfur variation.

Condition	Property	S60S-0.9	S60S-1.2	S60S-1.5	S80S-0.9	S80S-1.2	S80S-1.5
Temperature sweep (−80–70 ℃ under 0.2% strain)	E” at 0 ℃ [MPa]	25	25	31	45	49	50
	tan δ at 0 ℃	0.847	0.853	0.898	0.744	0.745	0.767
	tan δ at 60 ℃	0.112	0.108	0.101	0.134	0.123	0.120
Strain sweep (0.2–5% at 60 ℃)	E’ at 0.2% [Pa]	6079	6222	6423	11,788	12,439	13,000
	E’ at 5% [Pa]	4070	4255	4518	6096	6823	7423
	ΔE’ (0.2–5%) [Pa]	2009	1967	1906	5691	5616	5577

**Table 8:** Peak values of friction coefficient μ for vulcanizates from the rotational traction measuring system.

Compound	S60S-0.9	S60S-1.2	S60S-1.5	S80S-0.9	S80S-1.2	S80S-1.5
μ peak average (higher is better)	0.604	0.610	0.612	0.636	0.643	0.660
